# Association between indoor ventilation frequency and cognitive function among community-dwelling older adults in China: results from the Chinese longitudinal healthy longevity survey

**DOI:** 10.1186/s12877-022-02805-1

**Published:** 2022-02-07

**Authors:** Wenxin Wang, Jie Chen, Xurui Jin, Yongjing Ping, Chenkai Wu

**Affiliations:** 1 Department of Public Administration, Law School, Shantou University, Shantou, Guangdong China; 2 Institute of Local Government Development, Shantou University, Shantou, Guangdong China; 3 School of Public Health, Zhejiang University, Hangzhou, China; 4 MindRank AI ltd., Zhejiang, Hangzhou China; 5 Global Health Research Center, Duke Kunshan University, Academic Building 3038, No. 8 Duke Avenue, Kunshan, 215316 Jiangsu China

## Abstract

**Backgrounds:**

Emerging evidence suggested that indoor air pollution caused long-term adverse effects on cognitive function among older adults who spend more than 85% of their time indoors. Although high indoor ventilation can mitigate the effect of indoor air pollution on cognition among the younger population, limited evidence revealed the association between indoor ventilation and cognition among older adults.

**Methods:**

A total of 11,853 participants aged 65 and over (female, 54.5%; mean age, 83.6 years) were included from the 2017–2018 wave of the Chinese Longitudinal Healthy Longevity Survey (CLHLS). Indoor ventilation frequency was measured by the self-reported frequency of opening windows per week in each season. Cognitive function was evaluated by the Mini-Mental State Examination (MMSE). Multivariate Poisson regression with robust error variance was applied to assess the association between overall indoor ventilation frequency and cognitive function. We fitted in two adjusted models: Model 1 was adjusted for demographic; model 2 was further adjusted for lifestyle, socioeconomic status, health conditions, and environmental factors. The same models were also applied to measure the association between seasonal indoor ventilation frequency and cognitive function.

**Results:**

Of 11,853 older adults, 3035 (25.6%) had cognitive impairment. A high overall indoor ventilation frequency (indoor ventilation frequency: 6–8) was significantly associated with a 9% lower likelihood of cognitive impairment than low overall indoor ventilation frequency (indoor ventilation frequency: 0–3) among Chinese older adults [Relative risk (RR): 0.91, 95% Confidential Interval (CI): 0.83–0.99] in the full adjusted model. In the subgroup analysis in four seasons, high and intermediate indoor ventilation frequency in winter were significantly associated with 8% (RR: 0.92; 95%CI: 0.86–0.99) and 16% (RR: 0.84; 95%CI: 0.78–0.90) lower probability of cognitive impairment than low indoor ventilation frequency in the fully adjusted model among Chinese older adults, respectively.

**Conclusions:**

In this nationally representative cohort, a higher frequency of house ventilation by opening windows was significantly associated with a lower risk of cognitive impairment among Chinese older adults aged 65 and over. These results offered robust evidence for policymaking and health intervention to prevent older adults from cognitive impairment or dementia in developing regions.

**Supplementary Information:**

The online version contains supplementary material available at 10.1186/s12877-022-02805-1.

## Introduction

Cognitive impairment has been considered as the preclinical symptom of dementia [1, 2]. Cognitive impairment was a prevalent geriatric syndrome among older adults. In 2018, a systematic review showed that the prevalence of cognitive impairment, increasing with age, was 14.7% among Chinese adults aged 60 years and older [3]. Accumulating evidence demonstrated that air pollutants including particulate matter (PM), carbon monoxide (CO), sulfur and nitrogen oxides, methane, volatile organic compounds (VOCs) were detrimental to cognitive function among older adults [4–6]. In addition to outdoor air pollution, indoor air quality may have a substantial impact on individuals’ health, especially among older adults who spend around 85% of their time indoors [7–10]. Epidemiological studies showed that indoor air pollution caused by cooking fuels and incense burning was associated with poor cognitive performance among older adults [11, 12].

It has been documented that indoor ventilation, supplying air to indoor areas by natural or mechanical forces such as windows and air-conditioners, was able to mitigate the adverse effect of indoor air pollution on health among older adults [7, 13]. However, previous studies mainly revealed that a better house ventilation rate was associated with a lower risk of asthma and allergy symptoms, respiratory and lung diseases, and cognitive function among younger populations such as children and office workers in developed regions [7, 14, 15]. Few epidemiological investigations, to our knowledge, evaluated the association between indoor ventilation frequency and cognitive function among community-dwelling older adults.

China has the largest aging population in the world. The proportion of adults aged 60 years is projected to increase from 15.2% in 2015 to 25.3% in 2030 [16]. Therefore, it is imperative to evaluate the protective effect of indoor ventilation rate on cognitive function in the older population to provide practical evidence for policymaking and public interventions to promote healthy aging.

In the present cross-sectional study, we used the seasonal ventilation frequency data and the Mini-mental State Examination (MMSE) data from the 2017–2018 wave of Chinese Longitudinal Healthy Longevity Survey (CLHLS), including a large national representative sample aged over 65, to assess whether a higher indoor ventilation frequency is associated with better cognitive function among community-dwelling older adults in China.

## Methods

### Settings and participants

The present study used survey data from the 2017–2018 wave of the CLHLS. The CLHLS is an ongoing longitudinal study collecting extensive data among older adults aged 65 years and over since 1998 with biennial or triennial follow-ups. The CLHLS recruited participants from 631 randomly selected counties and cities in 22 out of 31 provinces. The sample represents approximately 85% of the total population in China. Detailed information on the CLHLS has been published elsewhere [17].

A total of 15,605 individuals participated in the 2017–2018 wave. We excluded 17 participants under 65 years old, 3427 participants with missing data on MMSE score, 234 participants with no indoor ventilation information. The final analytical sample included 11,853 participants. We did not use prior waves of the CLHLS because indoor ventilation was first measured in the 2017–2018 wave.

### Indoor ventilation

Indoor ventilation was measured by the self-reported weekly frequency of opening windows in each season. Participants were asked, “How often do you open windows for ventilation in spring, summer, fall, or winter?” We coded the response as follows: 0 point for 0 times/week, 1 point for 1–5 times/week, and 2 points for over 5 times/week. A total ventilation score was calculated by summing the indoor ventilation frequency of each season.

### Cognitive function

The Cognitive function in the CLHLS was measured by the Chinese version of the MMSE. A total of 24 items were included: orientation (4 points for time identification, 1 point for location recognition, 7 points for naming foods in 1 min), registration (3 points for reading 3 words without hesitation), attention and calculation (5 points for subtracting 3 continuously from 20, and 1 point for copying a figure), recall (3 points for delayed recall of 3 words listed in the registration), language (2 points for naming objectives, 1 point for repeating a sentence, and 3 points for following orders). The MMSE score ranged from 0 to 30 and a higher score represents a better cognitive function. We defined cognitive impairment as an MMSE score lower than 24 following previous research [18, 19].

### Covariates

We considered a variety of socio-demographic, lifestyle, environmental, and health characteristics as covariates. Socio-demographics included age (years), sex (male or female), years of education (0 years, 1–6 years, or more than 6 years), marital status (currently married and living with a spouse or others). Participants who were widowed, separated, divorced, or never married were merged into others due to the limited sample size. Family annual income (< 30,000 or ≥ 30,000) was measured as the annual income per capita of a participant’s household last year. Lifestyles included smoking status (non-current or current), alcohol drinking status (non-current or current), physical activity (regularly or not regularly), and body mass index (BMI; kg/m^2^) calculated as weight (kilograms) divided by height (meters) squared. Dietary diversity (low or high) measured the intake frequency of eight food types including vegetables, fruits, legume-related products, nuts, meat, eggs, fish, and dairy-related products. Participants who consumed more than four types of food were classified into high dietary diversity. Disability was evaluated by six Activities of Daily Living (ADLs) including bathing, dressing, toilet, indoor transfer, continence, and eating and classified into “not disabled” and “disabled”. Participants with at least one ADL disability were coded as “disabled”. Leisure activity was calculated by eight types of daily activities (Tai Chi, gardening, square dance, reading, playing cards, community social activities, watching TV or listening to the radio, feeding pets). Each leisure activity was scored as 1 for “never”, 2 for “sometimes”, and 3 for “almost every day”, we then summed the score from all leisure activities into an overall score for each participant. A total leisure activity score of 14 was considered impaired. Chronic diseases (diabetes, cancer, stroke, and cardiovascular disease) were self-reported. Additionally, we included the usage of cooking ventilation classified by the types of ventilation used in the kitchen when cooking at home (no or yes). Participants who used kitchen ventilation, fan, or opening window during cooking were coded as “yes”. Fuel choice was classified into clean fuel (natural gas, solar energy, and electricity) and polluted fuel (charcoal, firewood, or straw).

### Statistical analysis

We classified the indoor ventilation score into three categories (low:0–3; intermediate:4–5; high: 6–8). We used the Lowess smooth plot assessing the unadjusted association between continuous indoor ventilation score and cognitive impairment to determine the cut-off points of the indoor ventilation score (Additional file 1: Fig. 1). Baseline characteristics were presented using means and standard deviations (SD) for continuous variables and counts and percentages for categorical variables. Bivariate analyses were conducted by using the analysis of variance (ANOVA) for continuous variables and chi-square tests for categorical variables. Multivariable Poisson regression with robust error variance was used to assess the associations between the categorical indoor ventilation frequency and cognitive impairment. We fitted in two models: Model 1 included age and sex for adjustment; model 2 further adjusted residency, education level, marital status, body mass index, smoking status, drinking status, physical activity and dietary diversity, cooking ventilation, cooking fuel, and family income, leisure activity, the activity of daily living and chronic diseases. The linear regression models were also applied to evaluate the associations between the categorical indoor ventilation frequency and continuous MMSE score. In the subgroup analyses, the association between seasonal indoor ventilation frequency and cognitive impairment was assessed by multivariable Poisson regression with robust error variance for categorical cognitive function and linear regressions for continuous MMSE score. The adjusted models fitted in the same models aforementioned.

We calculated the relative risk (RR), beta coefficient, and 95% confidential interval (95%CI) to estimate the risk of categorical cognitive function and the difference in continuous cognitive function among different levels of indoor ventilation frequency.

All analyses were conducted using Stata/SE 15.0. A two-tailed *P*-value of less than 0.05 was considered statistically significant.

## Results

### Sample characteristics

A total of 11,853 participants were included in the statistical analysis. The mean age (SD) of the entire cohort was 83.6 (11.1) years and 6456 (54.5%) were female (Table 1). A total of 1133 (9.6%), 3858 (32.6%), and 6862 (57.9%) participants had a low (0–3), intermediate [4, 5], and high [6–8] indoor ventilation frequency, respectively. Participants with a higher indoor ventilation frequency were younger and married, more likely to have higher education levels, live in urban areas, and have higher family annual income, and were less likely to smoke, drink alcohol, and have high dietary diversity. Additionally, of those participants with a higher indoor ventilation frequency, 78.9% did not have any ADL disabilities, 47.9% did not have impaired leisure activities, 76.0% had a normal cognitive function, and 60.7% had regular physical activity. We did not find statistically significant differences in education, marital status, family annual income, or leisure activity in participants with and without missing data (Additional file 2: Table 1). For those covariates that showed a significant difference, the actual difference was not substantial, such as age (83.9 vs. 82.7 years) and sex (female: 53.7% vs. 56.8%).

**Table 1:** Baseline characteristics of included participants

Characteristics	Indoor ventilation frequency			Total ***N*** = 11,853 (100.0%)	***P***-value ^a^
	Low (0–3) ***N*** = 1133 (9.6%)	Intermediate (4–5) ***N*** = 3858 (32.6%)	High (6–8) ***N*** = 6862 (57.9%)		
Age, years, Mean (SD)	85.9 (11.0)	83.4 (11.0)	83.3 (11.1)	83.6 (11.1)	0.689
Female, Count (%)	646 (57.0)	2071 (53.7)	3739 (54.5)	6456 (54.5)	0.140
Education (years), Count (%)					< 0.001
None (0)	824 (72.7)	2235 (57.9)	3367 (49.1)	6426 (54.2)	
Primary school (1–6)	171 (15.1)	724 (18.8)	1437 (20.9)	2332 (19.7)	
Middle school or higher (> 6)	138 (12.2)	899 (23.3)	2058 (30.0)	3095 (26.1)	
Residence, Count (%)					< 0.001
Rural	663 (58.6)	1918 (49.8)	2644 (38.6)	5225 (44.1)	
Urban	468 (41.4)	1934 (50.2)	4209 (61.4)	6611 (55.9)	
Marital status, Count (%)					< 0.001
Married and living with spouse	725 (64.0)	2152 (55.8)	3806 (55.5)	6683 (56.4)	
Others	408 (36.0)	1706 (44.2)	3056 (44.5)	5170 (43.6)	
MMSE score, Count (%)					< 0.001
Severe (< 10)	99 (8.7)	198 (5.1)	371 (5.4)	668 (5.6)	
Moderate (10–18)	164 (14.5)	314 (8.1)	536 (7.8)	1014 (8.6)	
Mild (19–23)	167 (14.7)	447 (11.6)	739 (10.8)	1353 (11.4)	
Normal (> 23)	703 (62.0)	2899 (75.1)	5216 (76.0)	8818 (74.4)	
Depressive symptom, Count (%)					< 0.001
Without	325 (32.7)	1401 (39.0)	3195 (49.4)	4921 (44.5)	
With	670 (67.3)	2193 (61.0)	3270 (50.6)	6133 (55.5)	
Family annual income, Count (%)					< 0.001
< 30,000	704 (62.1)	1757 (45.5)	2475 (36.1)	4936 (41.6)	
≥30,000	429 (37.9)	2101 (54.5)	4387 (63.9)	6917 (58.4)	
Dietary diversity, Count (%)					< 0.001
Low	988 (88.3)	2910 (76.7)	4887 (71.8)	8785 (74.9)	
High	131 (11.7)	882 (23.3)	1924 (28.2)	2937 (25.1)	
Smoking, Count (%)					0.047
Non-current	970 (85.6)	3266 (84.7)	5714 (83.3)	9950 (83.9)	
Current	163 (14.4)	592 (15.3)	1148 (16.7)	1903 (16.1)	
Alcohol drinking, Count (%)					0.092
Non-current	977 (86.2)	3301 (85.6)	5786 (84.3)	10,064 (84.9)	
Current	156 (13.8)	557 (14.4)	1076 (15.7)	1789 (15.1)	
Physical activity, Count (%)					< 0.001
Regularly at present	892 (78.7)	2847 (73.8)	4166 (60.7)	7905 (66.7)	
Not regularly at present	241 (21.3)	1011 (26.2)	2696 (39.3)	3948 (33.3)	
BMI (kg/m^2^), mean (SD)	22.1 (3.8)	22.6 (3.8)	22.6 (3.8)	22.5 (3.8)	0.972
ADL condition, Count (%)					0.089
Not impaired	867 (76.5)	3069 (79.5)	5417 (78.9)	9353 (78.9)	
Impaired	266 (23.5)	789 (20.5)	1445 (21.1)	2500 (21.1)	
Leisure activity, Count (%)					< 0.001
Not impaired	342 (30.2)	1558 (40.4)	3288 (47.9)	5188 (43.8)	
Impaired	791 (69.8)	2300 (59.6)	3574 (52.1)	6665 (56.2)	

*Abbreviations*: *ADL* Activities of Daily Living, *BMI* Body Mass Index

^a^ *P*-values were calculated from chi-square tests or analysis of variance

### Association between indoor ventilation frequency and cognitive function

We found 3035 (25.6%) older adults being cognitively impaired (Table 2). Participants with cognitive impairment were associated with a higher proportion of the low indoor ventilation frequency than those with a normal cognitive function in each of four seasons among older adults (*p*-values < 0.001).

**Table 2:** Bivariate association between Seasonal ventilation frequency and cognitive function

Seasonal ventilation frequency	Cognitive function ^a^, Count (%)		***P***-value ^b^
	Normal ***N*** = 8818 (74.4)	Impaired ***N*** = 3035 (25.6)	
Spring ventilation frequency			< 0.001
0 time/week	358 (4.1)	242 (8.0)	
1–5 times/week	3521 (39.9)	1190 (39.2)	
> 5 times/week	4939 (56.0)	1603 (52.8)	
Summer ventilation frequency			< 0.001
0 time/week	198 (2.3)	140 (4.6)	
1–5 times/week	1847 (21.0)	691 (22.8)	
> 5 times/week	6773 (76.8)	2204 (72.6)	
Autumn ventilation frequency			< 0.001
0 time/week	315 (3.6)	215 (7.1)	
1–5 times/week	3402 (38.6)	1190 (39.2)	
> 5 times/week	5101 (57.9)	1630 (53.7)	
Winter ventilation frequency			< 0.001
0 time/week	1644 (18.6)	842 (27.7)	
1–5 times/week	3742 (42.7)	1116 (36.8)	
> 5 times/week	3412 (38.7)	1077 (35.5)	
Overall ventilation index			< 0.001
0–3 (low)	1874 (21.3)	833 (27.5)	
4–5 (intermediate)	2208 (25.0)	703 (23.2)	
6–8 (high)	4736 (53.7)	1499 (49.4)	

^a^ Any score of more than or equal to 24 indicates a normal cognition, and if the score of mini-mental state examination below this indicates cognitive impairment

^b^ *P*-values were calculated from chi-square tests or analysis of variance

We found a significant association between the overall indoor ventilation frequency (low, intermediate, and high) and cognitive impairment among older adults (Table 3). In the baseline model, the likelihood of cognitive impairment was 21% (RR: 0.79, 95%CI: 0.73–0.86) and 24% (RR: 0.76; 95%CI: 0.70–0.82) lower among older adults with intermediate and high indoor ventilation frequency than those with low overall indoor ventilation frequency, respectively. The associations attenuated but persisted to be significant in the fully adjusted model. Older adults with an intermediate and a high indoor ventilation frequency had 12% (RR: 0.88; 95%CI: 0.80–0.96) and 9% (RR: 0.91; 95%CI: 0.83–0.99) lower probability of cognitive impairment than those with a low indoor ventilation frequency, respectively. We did not find a significant interaction between fuel use in the kitchen and indoor ventilation rate (Additional file 3: Table 2). When the cognitive function was modeled as a continuous variable, older adults with an intermediate and a high indoor ventilation frequency had a 0.87-point (beta coefficient: 0.87; 95%CI: 0.45–1.29) and 0.74-point (beta coefficient: 0.74; 95%CI: 0.33–1.15) higher MMSE score than those with a low indoor ventilation frequency in the fully adjusted model, respectively (Table 4).

**Table 3:** The association between cognitive function and indoor ventilation frequency

	Low frequency	Intermediate frequency	High frequency
	RR (95%CI)		
Baseline Model ^a^	Ref.	0.79 (0.73–0.86)	0.76 (0.70–0.82)
Fully Adjusted Model ^b^	Ref.	0.88 (0.80–0.96)	0.91 (0.83–0.99)

*Abbreviation*: *RR* Relative risk, *CI* Confidence Interval

^a^ Baseline model: adjusted for age and sex

^b^ Fully adjusted model: adjust for age, sex, residency, education level, marital status, body mass index, smoking status, drinking status, physical activity and dietary diversity, cooking ventilation, cooking fuel, and family income, leisure activity, the activity of daily living, and chronic diseases (diabetes, heart disease, stroke, and cancer)

**Table 4:** The association between cognitive function (continuous) and indoor ventilation frequency

	Low frequency	Intermediate frequency	High frequency
	Beta Coefficients (95%CI)		
Baseline Model ^a^	Ref.	1.35 (0.96–1.73)	1.46 (1.09–1.82)
Fully Adjusted Model ^b^	Ref.	0.87 (0.45–1.29)	0.74 (0.33–1.15)

*Abbreviation*: *CI* Confidence Interval

^a^ Baseline model: adjusted for age and sex

^b^ Fully adjusted model: adjust for age, sex, residency, education level, marital status, body mass index, smoking status, drinking status, physical activity and dietary diversity, cooking ventilation, cooking fuel, and family income, leisure activity, the activity of daily living, and chronic diseases (diabetes, heart disease, stroke, and cancer)

### Association between seasonal indoor ventilation frequency and cognitive function

We found significant associations between indoor ventilation frequency and cognitive function among older adults in winter (Table 5). In winter, the probability of cognitive impairment among participants with an intermediate and a high frequency of indoor ventilation were 16% (RR: 0.84; 95%CI: 0.78–0.90) and 8% (RR: 0.92; 95%CI: 0.86–0.99) lower than those having low frequency of overall indoor ventilation. When cognitive function was measured as a continuous variable, the associations between indoor ventilation frequency and cognitive function reached statistical significant in spring (intermediate: beta coefficient: 0.84; 95%CI: 0.30–1.38; high: beta coefficient: 0.73; 95%CI: 0.19–1.27), autumn (intermediate: beta coefficient: 0.78; 95%CI: 0.22–1.34; high: beta coefficient: 0.70; 95%CI: 0.14–1.26) and winter (intermediate: beta coefficient: 0.69; 95%CI: 0.38–1.01; high: beta coefficient: 0.42; 95%CI: 0.09–0.74, Table 6).

**Table 5:** The association between cognitive function and seasonal indoor ventilation frequency

Season ^a^	Low frequency	Intermediate frequency	High frequency
	RR (95%CI)		
Spring	Ref.	0.87 (0.78–0.98)	0.92 (0.82–1.03)
Summer	Ref.	0.96 (0.82–1.12)	0.95 (0.82–1.10)
Autumn	Ref.	0.87 (0.78–0.98)	0.90 (0.80–1.01)
Winter	Ref.	0.84 (0.78–0.90)	0.92 (0.86–0.99)

*Abbreviations*: *RR* Relative risk, *CI* Confidence Interval

^a^ Fully adjusted model: adjusted for age, sex, residency, education level, marital status, body mass index, smoking status, drinking status, physical activity and dietary diversity, cooking ventilation, cooking fuel, and family income, leisure activity, the activity of daily living, and chronic diseases (diabetes, heart disease, stroke, and cancer)

**Table 6:** The association between cognitive function (continuous) and seasonal indoor ventilation frequency

Season ^a^	Low frequency	Intermediate frequency	High frequency
	Beta Coefficients (95%CI)		
Spring	Ref.	0.84 (0.30–1.38)	0.73 (0.19–1.27)
Summer	Ref.	0.26 (−0.45–0.97)	0.45 (− 0.24–1.13)
Autumn	Ref.	0.78 (0.22–1.34)	0.70 (0.14–1.26)
Winter	Ref.	0.69 (0.38–1.01)	0.42 (0.09–0.74)

*Abbreviation*: *CI* Confidence Interval

^a^ Fully adjusted model: adjusted for age, sex, residency, education level, marital status, body mass index, smoking status, drinking status, physical activity and dietary diversity, cooking ventilation, cooking fuel, and family income, leisure activity, the activity of daily living, and chronic diseases (diabetes, heart disease, stroke, and cancer)

## Discussion

In this large, national-representative cross-sectional study, we evaluated the association between overall and seasonal indoor ventilation frequency and cognitive impairment among Chinese older adults aged 65 and over, respectively. We found that more than 5 times using natural or mechanical house ventilation per week was significantly associated with a lower probability of cognitive impairment among older adults in the fully adjusted model. Furthermore, the significant association between indoor ventilation frequency and cognitive impairment retained in winter but attenuated in the other three seasons among the Chinese older population aged over 65. To our knowledge, the present study is among the first to evaluate the association between indoor ventilation frequency and cognitive function among the elderly aged over 65 in China.

Our findings were consistent with previous research showing that a high air ventilation rate was related to better health conditions among children and office workers [15, 20, 21]. For example, a double-blinded study recruiting 24 office workers spent 6 days on several environmentally controlled office spaces for cognitive tests [15]. Their results showed that a high air ventilation rate combined with high concentrations of VOCs was associated with better cognitive performance [15]. Another study analyzed the association between employees’ sick leave rate and air ventilation rate in a Massachusetts manufacturer [22]. Their results showed that a low air supply into the working buildings was related to increased sick leave, high risk of morbidity, and low productivity [22]. However, previous evidence focusing on employees and children only had a limited sample size in a single site and most of these studies were conducted in developed countries [20]. Our findings added new evidence to the older population in addition to children and employees, that a higher air ventilation rate was associated with better cognitive function. Furthermore, our study population had an extensive national outreach of the geographical regions and a national representative sample in China compared to previous investigations targeting on one site [13]. Therefore, our result may have better generalizability for the older population residence in other low- and middle-income countries (LMICs).

A high indoor ventilation frequency can reduce the risk of cognitive impairment among older adults in China may be attributed to the protective effect that air ventilation could reduce the concentration of air pollutants caused by cooking with solid fuels. The use of solid fuels was prevalent among Chinese households. In 2011, A large-scale cohort study randomly selected 91,527 volunteers/households throughout 31 provinces in China demonstrated that approximately 43.4% of Chinese households relied on solid fuels (biomass or coal) even though the proportion of solid fuels use declined in the last two decades [23]. Moreover, burning unpurified solid fuels such as coal on cooking stoves can produce a large amount of air pollutants including Sulfur Dioxides (SO_2_), Nitrogen oxides (NOx), CO, and PM, which can accumulate inside the households if the indoor air is not well ventilated [24, 25]. Additionally, The concentration of indoor PM was likely to rise by 1.5 to 27 times because of cooking-related activities [26]. The relation between PM and cognitive impairment among older adults has been well-documented previously, showing that a higher concentration of PM inside households was associated with a higher risk of cognitive impairment or dementia [4, 6, 27–29]. Therefore, since elders mainly spend time inside households, increasing ventilation frequency by opening windows or using air-conditioners is likely to be a protective strategy to control the indoor PM concentration and reduce the risk of cognitive impairment or dementia [7, 25].

The present study had several strengths. Firstly, we are among the first to assess the cross-sectional associations between indoor ventilation frequency and cognitive function among older adults in China. Second, we used an up-to-date and national representative cohort with a considerable sample size covering wide geographic regions in China. Third, we were able to control a variety of potential confounders with a comprehensive survey covering socio-demographic, environmental, and lifestyle factors. However, our study is not without limitations. First, the cross-sectional research design failed to capture the trends of cognitive decline and the variations of indoor ventilation rate in follow-up periods. Additionally, we could not rule out the possibility of reverse causality in a cross-sectional study. Further research using prospective cohort studies to investigate the incidence of cognitive impairment by indoor ventilation frequency is needed to further elucidate the causal relation. Second, the indoor ventilation in our study only measured the frequency of natural ventilation by opening windows. Other types of air ventilation such as mechanical ventilation including air-conditioning were not evaluated in our study due to the paucity of data in the CLHLS. Third, the ambient air pollutants may also affect the cognitive function among older adults since indoor ventilation may lead outdoor air pollutants into the individual residence. Previous observational studies on Chinese older adults revealed that exposure to ambient air pollutants such as PM2.5 or carbon monoxide (CO) was associated with poor cognitive performance [30–32]. Further observational studies on indoor ventilation and cognition could control outdoor air pollution in the multivariable analyses. Fourth, we may not fully control the heteroskedasticity and the ceiling effect in the linear regression model since more than 25% of participants had the highest MMSE score. Therefore, we dichotomized the MMSE score as our main outcome and the results of binary and continuous MMSE score showed consistency in our analyses.

## Conclusions

The present study firstly, to our knowledge, assessed the associations between overall and seasonal indoor ventilation frequency and cognitive function with a national representative large sample in China and revealed that a higher indoor ventilation frequency by opening windows more than five times per week was significantly associated with lower probability of cognitive impairment among elderly aged over 65. These findings added new and robust evidence of the protective effect of air ventilation on cognitive performance to the older population besides children and office workers. Further research is needed to assess the longitudinal association between indoor ventilation and cognitive decline among older adults with a long follow-up period and evaluate whether there are subgroups of the older population benefiting more from higher indoor ventilation frequency.

## Supplementary Information

**Additional file 1: Figure 1**. The Lowess smooth plot for the association between indoor ventilation frequency and cognitive function.

**Additional file 2: Table 1**. Descriptive statistics of participants with completed data and with missing data.

**Additional file 3: Table 2**. The association between indoor ventilation and cognition by fuel types.

## Data Availability

The datasets used in the present study are openly accessed in the Chinese Longitudinal Healthy Longevity Survey (CLHLS) from Peking University Open Research Data Platform. 10.18170/DVN/WBO7LK.

## Abbreviations

CLHLS: Chinese Longitudinal Healthy Longevity Survey
MMSE: Mini-Mental State Examination
OR: Odds ratios
CI: Confidential Interval
PM: Particulate matter
CO: Carbon monoxide
VOCs: Volatile organic compounds
BMI: Body mass index
ADLs: Activities of Daily Living
SD: Standard deviations
ANOVA: Analysis of variance
LMICs: Low- and middle-income countries
SO2: Sulfur Dioxides (SO2); NOx: Nitrogen oxides
