# Supplementary figures and images for: Association between indoor ventilation frequency and cognitive function among community-dwelling older adults in China: results from the Chinese longitudinal healthy longevity survey
Source: BMC Geriatr. 2022 Feb 7;22:106. doi: 10.1186/s12877-022-02805-1 (PMC8822634; doi:10.1186/s12877-022-02805-1)

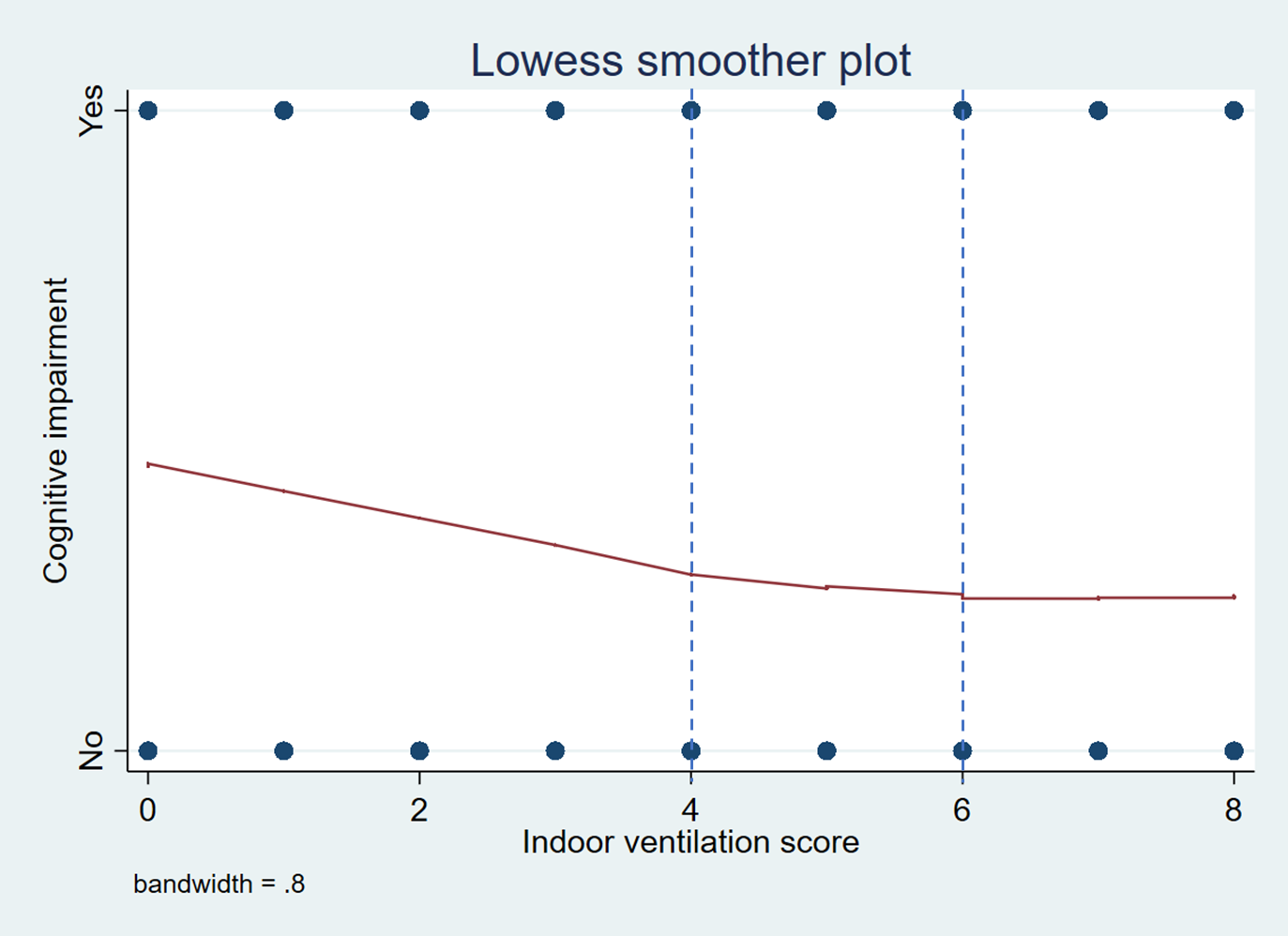

Supplement: Supplementary file 1 — Additional file 1: Figure 1. The Lowess smooth plot for the association between indoor ventilation frequency and cognitive function. [file 12877_2022_2805_MOESM1_ESM.docx]
