# Supplementary material for: Association between indoor ventilation frequency and cognitive function among community-dwelling older adults in China: results from the Chinese longitudinal healthy longevity survey
Source: BMC Geriatr. 2022 Feb 7;22:106. doi: 10.1186/s12877-022-02805-1 (PMC8822634; doi:10.1186/s12877-022-02805-1)
Supplement: Supplementary file 2 — Additional file 2: Table 1. Descriptive statistics of participants with completed data and with missing data. [file 12877_2022_2805_MOESM2_ESM.docx]

|  | **Completed data** | **With missing data** | **Total** | **P-value** |
| --- | --- | --- | --- | --- |
| **Characteristics** | **N=9044** | **N=2809** | **N=11853** |  |
| Age, years, Mean (SD) | 83.9 (11.2) | 82.7 (10.8) | 83.6 (11.1) | 0.018 |
| Female, Count (%) | 4860 (53.7) | 1596 (56.8) | 6456 (54.5) | 0.004 |
| Education (years), Count (%) |  |  |  | 0.119 |
| None (0) | 4939 (54.6) | 1487 (52.9) | 6426 (54.2) |  |
| Primary school (1-6) | 1785 (19.7) | 547 (19.5) | 2332 (19.7) |  |
| Middle school or higher (>6) | 2320 (25.7) | 775 (27.6) | 3095 (26.1) |  |
| Residence, Count (%) |  |  |  | <0.001 |
| Rural | 4073 (45.0) | 1152 (41.3) | 5225 (44.1) |  |
| Urban | 4971 (55.0) | 1640 (58.7) | 6611 (55.9) |  |
| Marital status, Count (%) |  |  |  | 0.625 |
| Married and living with spouse | 5088 (56.3) | 1595 (56.8) | 6683 (56.4) |  |
| Others | 3956 (43.7) | 1214 (43.2) | 5170 (43.6) |  |
| MMSE score, Count (%) |  |  |  | 0.017 |
| Severe (<10) | 533 (5.9) | 135 (4.8) | 668 (5.6) |  |
| Moderate (10-18) | 746 (8.2) | 268 (9.5) | 1014 (8.6) |  |
| Mild (19-23) | 1051 (11.6) | 302 (10.8) | 1353 (11.4) |  |
| Normal (>23) | 6714 (74.2) | 2104 (74.9) | 8818 (74.4) |  |
| Depressive symptom, Count (%) |  |  |  | 0.025 |
| Without | 3794 (45.1) | 1127 (42.6) | 4921 (44.5) |  |
| With | 4616 (54.9) | 1517 (57.4) | 6133 (55.5) |  |
| Family annual income, Count (%) |  |  |  | 0.992 |
| <30,000 | 3766 (41.6) | 1170 (41.7) | 4936 (41.6) |  |
| ≥30,000 | 5278 (58.4) | 1639 (58.3) | 6917 (58.4) |  |
| Dietary diversity, Count (%) |  |  |  | 0.006 |
| Low | 6832 (75.5) | 1953 (72.9) | 8785 (74.9) |  |
| High | 2212 (24.5) | 725 (27.1) | 2937 (25.1) |  |
| Smoking, Count (%) |  |  |  | 0.002 |
| Non-current | 7540 (83.4) | 2410 (85.8) | 9950 (83.9) |  |
| Current | 1504 (16.6) | 399 (14.2) | 1903 (16.1) |  |
| Alcohol drinking, Count (%) |  |  |  | 0.040 |
| Non-current | 7645 (84.5) | 2419 (86.1) | 10064 (84.9) |  |
| Current | 1399 (15.5) | 390 (13.9) | 1789 (15.1) |  |
| Physical activity, Count (%) |  |  |  | 0.010 |
| Regularly at present | 6088 (67.3) | 1817 (64.7) | 7905 (66.7) |  |
| Not regularly at present | 2956 (32.7) | 992 (35.3) | 3948 (33.3) |  |
| BMI (kg/m^2^), mean (SD) | 22.0 (3.5) | 24.3 (4.1) | 22.5 (3.8) | <0.001 |
| ADL condition, Count (%) |  |  |  | <0.001 |
| Not impaired | 7207 (79.7) | 2146 (76.4) | 9353 (78.9) |  |
| Impaired | 1837 (20.3) | 663 (23.6) | 2500 (21.1) |  |
| Leisure activity, Count (%) |  |  |  | 0.071 |
| Not impaired | 4000 (44.2) | 1188 (42.3) | 5188 (43.8) |  |
| Impaired | 5044 (55.8) | 1621 (57.7) | 6665 (56.2) |  |
