# Supplementary material for: Association between indoor ventilation frequency and cognitive function among community-dwelling older adults in China: results from the Chinese longitudinal healthy longevity survey
Source: BMC Geriatr. 2022 Feb 7;22:106. doi: 10.1186/s12877-022-02805-1 (PMC8822634; doi:10.1186/s12877-022-02805-1)
Supplement: Supplementary file 3 — Additional file 3: Table 2. The association between indoor ventilation and cognition by fuel types. [file 12877_2022_2805_MOESM3_ESM.docx]

| **Indoor ventilation** | **Clean fuels** | **Polluted fuels** | **P-value for interaction ^a^** |
| --- | --- | --- | --- |
|  | **RR (95%CI)** | |  |
| Low | Ref. | Ref. |  |
| Intermediate | 0.86 (0.77 - 0.97) | 0.89 (0.77 - 1.02) | 0.548 |
| High | 0.90 (0.81 - 1.01) | 0.89 (0.78 - 1.02) | 0.753 |
